# Postpartum Depression, Pain Catastrophizing, and Coping Strategies in Early Postpartum Women

**DOI:** 10.1192/j.eurpsy.2025.1890

**Published:** 2025-08-26

**Authors:** O. Bouattour, N. Messedi, F. Khanfir, R. Ben Jmeaa, I. Chaari, F. Charfeddine, L. Aribi, K. Chaabene, J. Aloulou

**Affiliations:** 1 Psychiatry « B » department; 2 Gynecology department, Hedi Chaker University Hospital, Sfax, Tunisia

## Abstract

**Introduction:**

The postnatal period is a time of great vulnerability in terms of mental health, with depression being one of the most common complications. This condition can significantly affect how women perceive and process the pain and stress associated with childbirth. Pain experienced during pregnancy and postpartum is linked to psychological distress, often influenced by pain catastrophizing a cognitive tendency to dwell on, magnify, or feel helpless in the face of pain. To manage these challenges, many women rely on coping mechanisms to navigate the significant stressors of this period.

**Objectives:**

The aim of this study is to explore the relationship between postpartum depression, pain catastrophizing, and coping mechanisms in the postnatal period.

**Methods:**

We conducted a cross-sectional descriptive and analytical study targeting women in their first week postpartum who had been admitted to the gynaecology-obstetrics department of the Hedi Chaker University Hospital in Sfax, Tunisia. The study was conducted over a three-month period (October, November and December 2023). We used the Tunisian Arabic version of the Edinburgh Postnatal Depression Scale (EPDS). Pain catastrophisation was assessed using the pain catasrophizing scale (PCS). We used the French version of the coping scale Ways of coping checklist revised (WCC) to evaluate coping strategies.

**Results:**

The study included 220 postpartum women with a mean age of 31.1 ± 6.6. Psychiatric history was recorded in 5.5% of participants, predominantly bipolar disorders (4.1%). Medical or surgical history was reported by 14.1% of women. Among the participants, 28.6% were primiparous, and 71.4% were multiparous. A history of child loss was noted in 4.5% of cases. Spontaneous labor occurred in 65.5% of women, while 17.7% underwent induced labor. Vaginal deliveries were performed in 56.4% of cases, with forceps used in 8.2%. Postpartum recovery was uncomplicated for 86.4% of participants, while complications occurred in 13.6% of cases. Postnatal care was provided by family members for 55% of women. Postpartum depression was observed in 20.9% of participants.The average score of Pain Catastrophizing Scale (PCS) was 24 ± 11, and problem-focused coping was the most frequently employed strategy, with a mean score of 26.51 ± 6.3.

Women with postpartum depression had significantly higher PCS scores (p<0,001). Emotion-focused coping was the predominant strategy used by this group (p=0.003). Conversely, women without postpartum depression were more likely to use problem-focused coping (p<0.001) and social support-based coping (p=0.011).

**Conclusions:**

This study reveals that postpartum depression is associated with higher pain catastrophizing and a greater use of emotion-focused coping. In contrast, women without depression tended to use problem-focused and social support-based coping, suggesting that these strategies may help mitigate postpartum psychological distress.

**Disclosure of Interest:**

None Declared

